# Disruption of BCAA degradation is a critical characteristic of diabetic cardiomyopathy revealed by integrated transcriptome and metabolome analysis

**DOI:** 10.1515/biol-2022-0974

**Published:** 2024-12-31

**Authors:** Yanxia Wu, Wanxiang Jiang, Junlong Wang, Guoqing Xie, Yan Sun, Jinliang Yang

**Affiliations:** State/National Key Laboratory of Biotherapy, Sichuan University, Chengdu, Sichuan, 610000, P. R. China; Sichuan Greentech Bioscience Co., Ltd., Meishan, Sichuan, 620010, P. R. China

**Keywords:** diabetic cardiomyopathy, multi-omics, amino acid metabolism, branched-chain amino acids

## Abstract

In this study, we integrated transcriptomic and metabolomic analyses to achieve a comprehensive understanding of the underlying mechanisms of diabetic cardiomyopathy (DCM) in a diabetic rat model. Functional and molecular characterizations revealed significant cardiac injury, dysfunction, and ventricular remodeling in DCM. A thorough analysis of global changes in genes and metabolites showed that amino acid metabolism, especially the breakdown of branched-chain amino acids (BCAAs) such as valine, leucine, and isoleucine, is highly dysregulated. Furthermore, the study identified the transcription factor Gata3 as a predicted negative regulator of the gene encoding the key enzyme for BCAA degradation. These findings suggest that the disruption of BCAA degradation is a critical characteristic of diabetic myocardial damage and indicate a potential role for Gata3 in the dysregulation of BCAA metabolism in the context of DCM.

## 1 Introduction

Heart failure (HF) is a major cause of death and illness worldwide [1]. According to the World Health Organization, HF affects about 1–2% of adults in developed countries, with a higher prevalence among the elderly (10% of men and 8% of women over 60) [2,3]. Diabetic cardiomyopathy (DCM), first identified in the 1970s, is a specific type of cardiomyopathy seen in diabetic patients without hypertension, coronary atherosclerosis, or valvular disease [4]. The progression of DCM involves several mechanisms that contribute to heart dysfunction. Metabolic issues, such as poor glucose utilization and abnormal fatty acid metabolism, play a key role in DCM development [5]. These metabolic problems lead to an energy imbalance in heart cells, causing poor contractile function and structural changes. Oxidative stress, defined as an imbalance between reactive oxygen species (ROS) production and antioxidant defenses, is another important factor in DCM development [6]. Increased ROS production in diabetes can damage cell components like lipids, proteins, and DNA, leading to cell dysfunction and death [7]. Myocardial fibrosis, characterized by excessive collagen and other extracellular matrix deposits, is a hallmark of DCM. Fibrosis disrupts the normal structure of the heart muscle, affecting its relaxation and contraction, and leads to diastolic dysfunction [8]. Cardiac hypertrophy, or the increase in size and mass of heart muscle cells, is also common in DCM. This hypertrophic response is driven by factors such as insulin resistance, inflammation, and neurohormonal activation [9].

Several medications have been used to target the pathophysiological mechanisms in DCM, mainly to control high blood sugar and restore heart function. These include antidiabetic drugs, antioxidants, and agents that affect myocardial fibrosis and hypertrophy [10]. Despite these treatments, there is a persistent increase in morbidity and mortality rates related to DCM, highlighting the need for further research. A thorough understanding of the fundamental mechanisms of DCM is crucial for developing more effective treatment strategies.

Advancements in molecular biology technology have led to the development of high-throughput techniques such as transcriptomics and metabolomics. These methods provide valuable strategies for understanding the complex pathogenesis of diseases [11,12].

In this study, we developed a DCM animal model using streptozotocin (STZ) and conducted multi-omics profiling of myocardial tissue. We performed transcriptome analysis to examine changes in RNA transcripts, which helped us identify the genes and pathways involved in DCM progression. Additionally, we conducted metabolome analysis to explore the global metabolic profile and identify metabolic abnormalities associated with DCM. By integrating transcriptomics and metabolomics data, we aimed to gain a comprehensive understanding of the underlying mechanisms of DCM, paving the way for further research and the development of therapeutic interventions.

## 2 Materials and methods

### 2.1 Model preparation

The animal protocols used in this study were reviewed and approved by the Animal Care and Use Committee of Sichuan Greentech Bioscience Co., Ltd., China, ensuring compliance with ethical guidelines for animal research. Male Sprague-Dawley (SD) rats, aged 6–8 weeks, were obtained from Sipeifu (Beijing, China) Experimental Animal Technology Co., Ltd. The rats were housed in a controlled environment with a consistent 12-hour light–dark cycle, a temperature range of 20–26°C, and humidity levels between 40 and 70%. They had unrestricted access to water and standard rodent chow. After a one-week acclimatization period, the rats were randomly divided into two groups: a control group and a model group. Diabetes was induced in the model group with a single intraperitoneal injection of STZ at a dose of 65 mg/kg, diluted in citrate buffer, after an overnight fast. The control group received an equivalent volume of citrate buffer. Fasting venous blood samples were collected from the tail of each rat on days 3 and 7 after STZ injection. Rats in the model group with blood glucose levels exceeding 11.1 mmol/L were considered diabetic and included in the study. This ensured that the model group accurately represented the diabetic condition for further analysis of DCM development compared to the control group.


**Ethical approval:** The research related to animal use has been complied with all the relevant national regulations and institutional policies for the care and use of animals and has been approved by the Animal Care and Use Committee of Sichuan Greentech Bioscience Co., Ltd., China.

### 2.2 Echocardiographic examinations

During anesthesia, cardiac contraction and relaxation were noninvasively assessed using transthoracic echocardiography, with isoflurane administered via a mask at the minimum effective concentration. Parasternal long- and short-axis images were obtained 5 months after STZ or placebo injection. Standard echocardiographic techniques were used to acquire two-dimensional, M-mode, and color-guided pulsed-wave Doppler images. The following parameters were carefully measured: left ventricular internal dimension at end diastole (LVIDd), left ventricular internal dimension at end systole (LVIDs), interventricular septal thickness at end diastole (IVSd), interventricular septal thickness at end systole (IVSs), left ventricular posterior wall thickness at end diastole (LVPWd), left ventricular posterior wall thickness at end systole (LVPWs), left ventricle end-diastolic volume (LVEDV), left ventricle end-systolic volume (LVESV), left ventricular fractional shortening (FS), ejection fraction (EF), wave *E* deceleration time (DT), isovolumic diastole time (IVRT), *E*/*A* ratio, and *E*/*e*′ ratio. Each parameter was measured three times by a single blinded observer to ensure accuracy and reproducibility.

### 2.3 Biochemical analysis

At the end of the study, serum samples were collected from all experimental rats. The concentrations of cholesterol (CHOL), high-density lipoprotein cholesterol (HDL-C), low-density lipoprotein cholesterol (LDL-C), triglycerides (TRIG), lactate dehydrogenase (LDH), and creatine kinase-MB (CK-MB) were then measured using an automated biochemistry analyzer.

### 2.4 Hemodynamics

The rats were anesthetized with an intraperitoneal injection of 3% pentobarbital sodium at a dose of 60 mg/kg. To maintain a consistent body temperature of 37°C, the animals were placed on regulated heating pads, and their core temperature was continuously monitored with a rectal probe. A polyethylene catheter was inserted into the left ventricle through the right jugular artery. After a stabilization period of 5 min, physiological signals were recorded using a multi-channel physiological signal system equipped with a pressure transducer. The recorded parameters included heart rate (HR), left ventricular systolic pressure (LVSP), left ventricular diastolic pressure (LVDP), the maximum rate of pressure rise during systole (+d*p*/d*t*_max_), and the maximum rate of pressure decline during diastole (−d*p*/d*t*_min_).

### 2.5 Determination of heart weight

Five months after diabetes induction, the animals were humanely euthanized, and their hearts were carefully removed. The hearts were dried using filter paper to remove any residual moisture before being weighed. The heart weight of each rat was then recorded.

### 2.6 Histopathology

Heart tissues from six rats in each group were preserved in a 10% formalin solution. These specimens were then embedded in paraffin and sectioned into 5 μm thick slices. The slides were stained with hematoxylin-eosin (H&E) and Masson’s trichrome stains. Wheat germ agglutinin (WGA) staining was also used to assess cardiocyte size. Following the manufacturer’s guidelines, the WGA-stained sections were examined by a blinded expert using a microscope. Cardiocyte dimensions were measured using ImageJ software, with five sections per rat analyzed for quantification.

### 2.7 RNA sequencing (RNA-seq) data analysis

RNA was extracted from heart tissues using TRIzol (Thermo Fisher Scientific, USA), and sequencing libraries were prepared using the NEBNext^®^ Ultra™ RNA Library Prep Kit for Illumina^®^ (NEB, USA). The libraries were purified using AMPure XP beads and sequenced on an Illumina Novaseq platform. Differentially expressed genes (DEGs) were identified using the DESeq2 algorithm in the DESeq2 R package, with genes having a |log2FC| > 1 and a *P*-value of <0.05 considered differentially expressed. A volcano plot was generated using *R*, with colors indicating the filtering criteria. Additionally, Gene Ontology (GO) and KEGG pathway enrichment analyses, along with cluster analysis of DEGs, were performed using the Wekemo Bioincloud platform (https://www.bioincloud.tech). The iRegulon plugin in Cytoscape software (v3.10.0) was used to predict transcription factors (TFs) for key genes in critical pathways, and TFs with significant expression patterns were selected.

### 2.8 Metabonomic analysis

The non-targeted metabolomics of the heart in both control and model groups were examined using liquid chromatography-mass spectrometry. Differentially altered metabolites (DAMs) identified in both positive and negative ion modes were analyzed using Student’s *t*-tests. A partial least squares discriminant analysis (PLS-DA) was then conducted, with significance thresholds set at |Log2FC| > 1, PLS-DA VIP > 1, and *P*-value <0.05. Significant DAMs were visualized using volcano plots and heat maps. To further understand the biological pathways associated with these DAMs, a KEGG pathway analysis was performed using the Wekemo Bioincloud platform (https://www.bioincloud.tech).

### 2.9 Integrated analysis of transcriptome and metabolome

Genes and metabolites with significant differences were analyzed using the Pearson correlation coefficient to assess the relationship between DEGs and DAMs. To explore the underlying mechanisms, all DEGs and DAMs were integrated into MetaboAnalyst (version 5.0, www.metaboanalyst.ca) for joint-pathway analysis, identifying the targeted pathways. Key biochemical and signal transduction pathways were mapped, integrated, and visualized using Pathview Web and MetScape.

### 2.10 Identification of TFs that regulated the critical pathway

The Cytoscape plugin iRegulon was used to analyze TFs regulating hub genes. iRegulon identifies regulons using motifs and tracks discovery within an existing network or a set of regulated genes. The cutoff criteria were: enrichment score threshold = 3.0, ROC threshold for AUC calculation = 0.03, rank threshold = 5,000, minimum identity between orthologous genes = 0.0, and FDR = 0.001.

### 2.11 Statistical analysis

Data were analyzed using Stata v15.0 software, with results presented as mean ± standard deviation (*x̅* ± SD). Comparisons between the two groups were conducted using the Student’s *t*-test, with statistical significance defined as a *P*-value of less than 0.05.

## 3 Results

### 3.1 The DCM rats present with systolic and diastolic dysfunction, as well as ventricular remodeling

**Figure 1:**
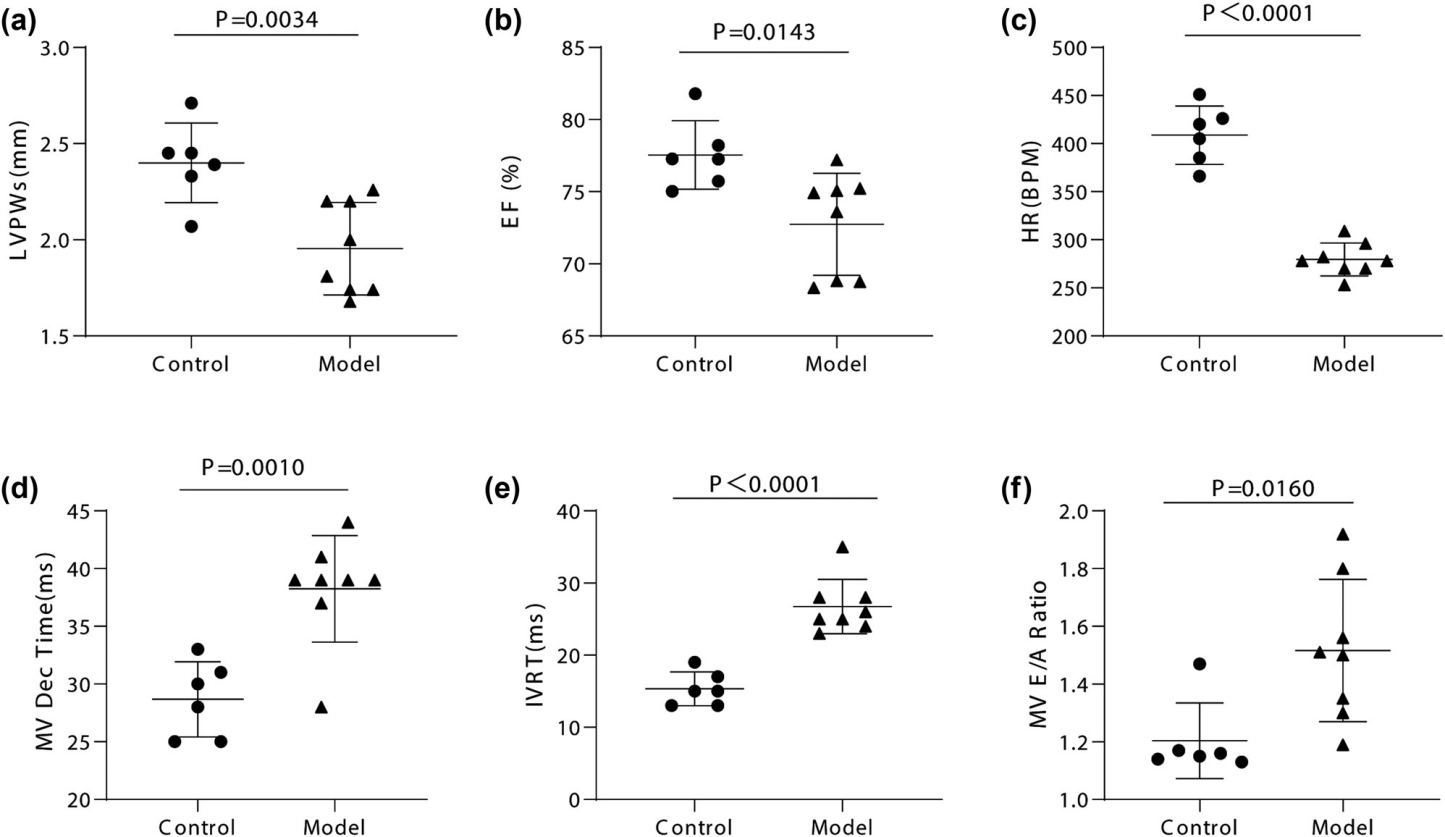
DCM resulted in left ventricular diastolic and systolic dysfunction by echocardiography analysis. (a–c) Measurement in M-mode of left ventricular EF, left ventricular posterior wall (LVPW), and heart rate (HR). (d–f) Measurement in Doppler echocardiography of *E*/*A* ratio, Isovolumic relaxation time (IVRT), and DT (*n* = 6–8).

Transthoracic echocardiography was performed 5 months after the onset of diabetes to evaluate its impact on cardiac structure and function. The results, shown in Table S1 and Figure 1, indicate significant changes in the diabetic rats compared to the control group.

Diabetic rats exhibited significantly reduced posterior wall thicknesses (LVPWs and LVPWd) compared to normal rats. Additionally, echocardiography revealed a decline in the systolic function of the LV in the model group, demonstrated by notably lower EF and FS values.

Moreover, there was a significant reduction in *E*, *A*, and *e*′ velocities in diabetic rats compared to normal rats, indicating impaired diastolic function. The *E*/*A* and *E*/*e*′ ratios were significantly increased in diabetic rats, further highlighting diastolic dysfunction. Additionally, diabetic rats showed prolonged mitral valve (MV) DT and isovolumic relaxation time (IVRT), suggesting impaired left ventricular relaxation.

Interestingly, the HR in diabetic rats was significantly lower than in normal rats. However, no significant differences were found between the groups in interventricular septum thicknesses (IVSs and IVSd), left ventricular diameters (LVIDs and LVIDd), and left ventricular volumes (LVESV and LVEDV).

These findings underscore the significant impact of diabetes on cardiac structure and function, evidenced by reduced posterior wall thickness, impaired systolic function, and compromised diastolic function. The prolonged MV DT and IVRT suggest a deficiency in left ventricular relaxation. The lower HR observed in diabetic rats may be a compensatory response to these cardiac dysfunctions.

### 3.2 The diabetic rats exhibit impaired systolic function, relaxation, and increased ventricular pressure in hemodynamics

In terms of hemodynamics, DCM rats showed significantly lower LVSP (106.9 ± 7.54 mmHg vs 156.33 ± 22.8 mmHg, *P* < 0.01), maximum rate of pressure rise (+d*p*/d*t*_max_) (2740.34 ± 241.02 mmHg/s vs 4764.78 ± 650.36 mmHg/s, *P* < 0.01), and maximum rate of pressure decline (−d*p*/d*t*_min_) (2199.68 ± 162.01 mmHg/s vs 4324.01 ± 550.69 mmHg/s, *P* < 0.01) compared to the control group. Additionally, a significantly higher LVDP was observed in the model group (Figure S1). These results indicate that diabetes and hyperglycemia impair systolic function, relaxation, and increase ventricular pressure, which may contribute to the development and progression of DCM.

### 3.3 The DCM rats demonstrate elevated serum cardiac injury markers and dyslipidemia

The DCM rats also showed elevated serum cardiac injury markers and dyslipidemia. Serum levels of LDH and CK-MB were significantly higher in DCM rats compared to control rats (Figure S2a and b), suggesting myocardial damage due to diabetes. Furthermore, plasma lipid levels were altered; total CHOL, triglycerides, and LDL-C were significantly higher in DCM rats than in normal rats (Figure S2c–e), while there was no significant difference in HDL-C between the two groups (Figure S2f).

### 3.4 The DCM rats demonstrate structural deficits in heart

**Figure 2:**
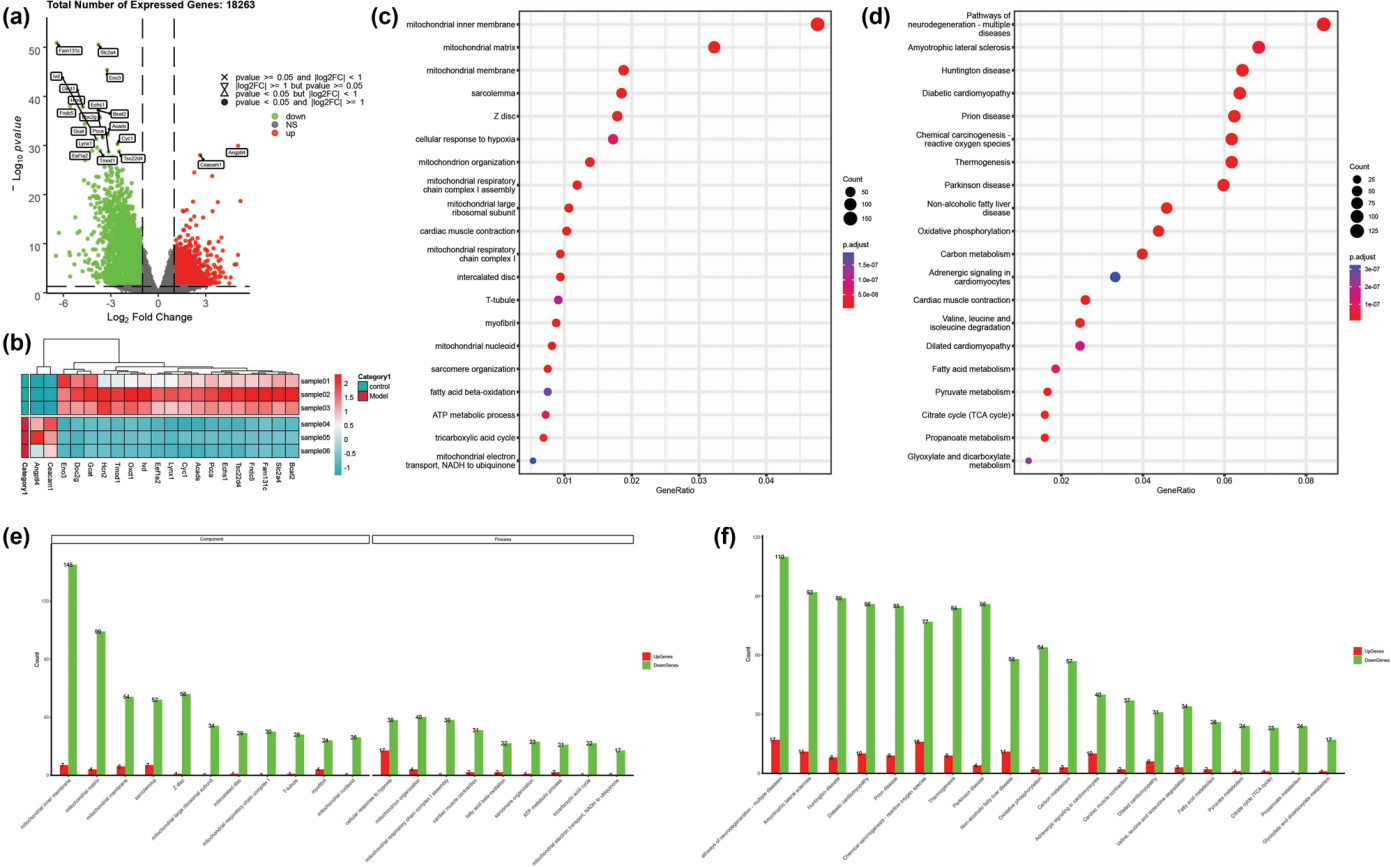
The results of RNA-seq sequence analyses of heart. (a) The volcano plot showing the distribution of log2 FC and *P* values for the differential expressed genes of the hearts in response to hyperglycemia. Red dots, significantly up-regulated genes; green dots, significantly down-regulated genes; grey triangles, no significant difference. (b) The heat map of top 20 DEGs. The expression level of genes in different samples is represented by different colors. The scale at the right denoted normalized expression levels (red, high expression; blue, low expression). (c) Dot plot of the top 20 GO terms in GO analysis of DEGs. (d) The 20 top KEGG pathways in the KEGG analysis of DEGs. (e) The numbers of up-regulated and down-regulated genes in the top 20 GO terms. (f) The numbers of up-regulated and down-regulated genes in the top 20 KEGG pathways.

The DCM rats also showed structural deficits in the heart. After hemodynamic measurements, the hearts were removed and weighed. As shown in Figure S3b, the heart weight of DCM rats was significantly reduced five months after STZ-induced diabetes. H&E staining of heart tissues in the control group showed regular myocardial fibers and normal morphology of cardiac myocytes (Figure 2a). However, in the diabetic model group, there was slight myocardial cell death and a mild increase in inflammatory cells in the myocardial interstitium, characterized by scattered distribution of eosinophilic enhanced and condensed nuclei in myocardial cells and increased inflammatory cells within the capillaries. Masson’s trichrome staining showed no obvious increased collagen deposition in heart tissues. Measurement of cell size in transverse WGA-stained sections revealed decreased cardiomyocyte size in diabetic rats. Figure S3a shows representative images of myocytes stained with WGA in normal and DCM rats, and Figure S3c shows the statistical results. The analysis revealed that DCM caused smaller myocytes compared to normal rats (472.06 ± 38.39 μm² in DCM vs 587 ± 25.64 μm² in normal, *P* < 0.001).

### 3.5 Transcriptome analysis reveals significant differences in gene expression between DCM and normal rats

Applying the cutoffs of *P*_adj_ < 0.05 and |log2| FC > 1, we identified 3,880 DEGs between the normal and model groups. Among these, 1,593 genes were significantly up-regulated, while 2,287 genes were significantly down-regulated (Figure 2a and Table S3). Notably, among the 3,880 DEGs, 175 TFs were identified, with 94 TFs significantly up-regulated and 81 significantly down-regulated (Table S3). To illustrate the expression patterns of these DEGs, a heat map and hierarchical clustering were constructed, with the top 20 DEGs shown in Figure 2b. The heat map depicted gene expression levels across different samples using colors ranging from light blue (low expression) to dark red (high expression), highlighting distinct gene expression patterns between the control and model groups.

**Figure 3:**
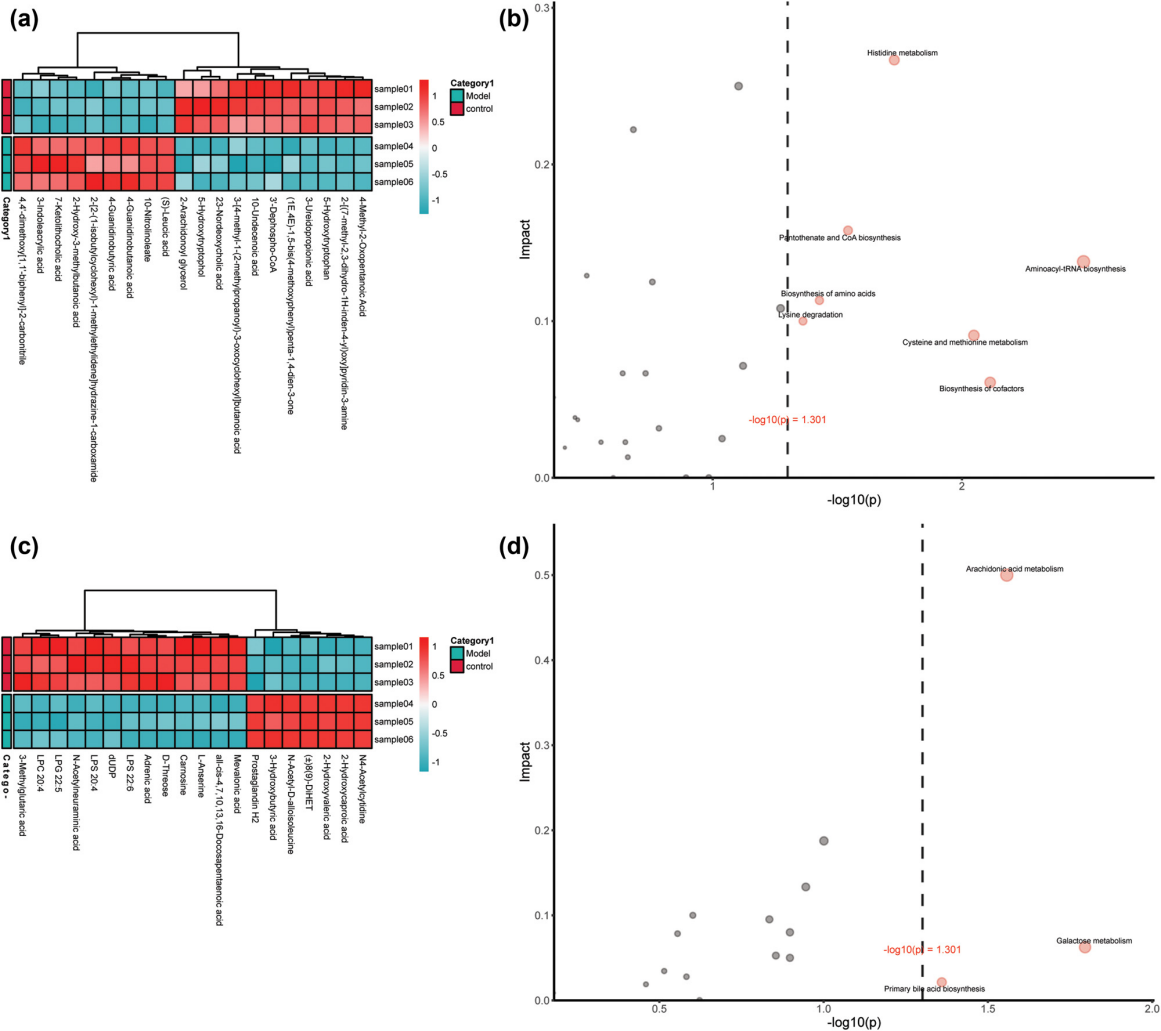
The function enrichment and hierarchical clustering of the differential metabolites. (a and b) Heat map of the top 20 differential metabolites in positive mode and negative mode. (c and d) The top 20 KEGG pathway enrichment and topology analysis results of positive mode and negative mode.

GO analysis revealed that 540 terms were significantly enriched (*P* < 0.05) within the DEGs, categorized into biological process, cellular component, and molecular function groups, comprising 380, 66, and 94 terms, respectively (Table S4). Among the top 20 most significantly enriched GO terms, as shown in Figure 2c, several are associated with mitochondrial structure or biological processes. These include the mitochondrial inner membrane, mitochondrial matrix, mitochondrial large ribosomal subunit, mitochondrial respiratory chain complex I, assembly of mitochondrial respiratory chain complex I, tricarboxylic acid cycle, ATP metabolic process, and mitochondrial electron transport. Figure 3d reveals that the top 20 most significantly enriched GO terms were down-regulated, indicated by the down-regulation of most genes in these pathways.

The KEGG pathway enrichment analysis identified 82 significantly enriched pathways between the control and model groups (Table S5). There was a notable enrichment of DEGs in carbohydrate metabolism, energy metabolism, and amino acid metabolism. The top 20 KEGG pathways, mainly related to metabolic processes, included carbon metabolism, the citrate cycle (TCA cycle), pyruvate metabolism, glyoxylate and dicarboxylate metabolism, propanoate metabolism, oxidative phosphorylation, valine, leucine, and isoleucine degradation, as well as fatty acid metabolism (Figure 2d). Additionally, DEGs were enriched in pathways related to DCM, cardiac muscle contraction, and dilated cardiomyopathy. Figure 3d shows that the top 20 most significantly enriched KEGG pathways were down-regulated, indicated by the down-regulation of most genes in these pathways.

### 3.6 Metabolomic analysis reveals metabolite perturbations between in DCM rats

Metabolomic profiles were evaluated using PLS-DA, revealing a clear distinction between the control and model groups in both positive and negative modes (Figure S4). To identify differentially accumulated metabolites, the criteria of VIP > 1, *P* < 0.05, and fold change >2 were used. Based on these criteria, 53 metabolites were identified in the positive ion mode and 56 in the negative ion mode. In the positive ion mode, 30 metabolites were down-regulated in diseased tissues, while 23 metabolites were up-regulated. In the negative ion mode, 26 metabolites were up-regulated, and 30 metabolites were down-regulated (Table S6).

To visualize the overall changes in the heart metabolome, we constructed a clustered heat map of DAMs. This heat map provides a detailed view of the metabolomic alterations between the model and normal groups (Figure 3c and d). Subsequent analysis revealed that these DAMs were predominantly enriched in categories such as amino acids, peptides and analogs, fatty acids and conjugates, carbohydrates and carbohydrate conjugates, and glycerophosphocholines (Table S2).

To elucidate the pathways associated with the differentially abundant metabolites, we performed enrichment and topological analyses. These analyses revealed significant enrichment in multiple pathways. In the positive ion mode, notable enrichment was observed in pathways such as aminoacyl-tRNA biosynthesis, cofactor and cysteine metabolism, histidine metabolism, pantothenate and CoA biosynthesis, amino acid biosynthesis, and lysine degradation. In the negative ion mode, significant enrichment was found in galactose metabolism, arachidonic acid metabolism, and primary bile acid biosynthesis pathways (Figure 3a and b, Table S7).

### 3.7 Integrated analysis of transcriptome and metabolome reveals disrupted branched-chain amino acids (BCAAs) degradation in DCM rats

**Figure 4:**
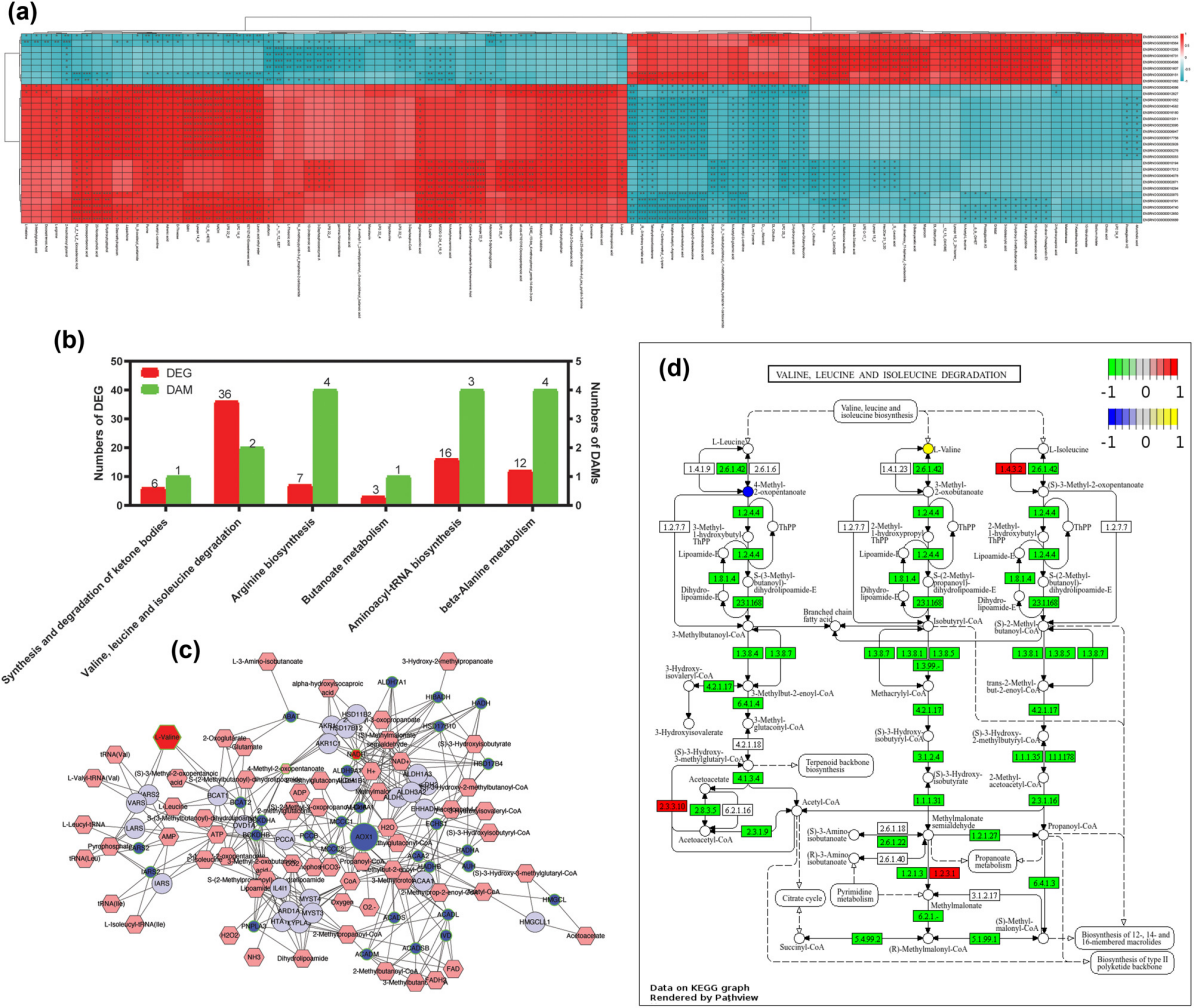
Integrated analysis of transcriptome and metabolome, (a) The top 50 heat plot of the correlations between metabolites (columns) and genes (rows). The red and blue colors show the positive and negative correlations between transcriptomics and metabolomics data (*P < 0.05, **P < 0.01, ***P < 0.05). (b) Numbers of DEGs and metabolites in comapped significant enrichment pathways by joint KEGG enrichment analysis. (c) Schematic diagram of the valine, leucine, and isoleucine degradation pathway, the red hexagonal boxes with green borders represent the DAMs, with larger boxes indicating up-regulated DAMs and smaller boxes representing down-regulated DAMs. The blue circles with green borders represent the DEGs, with larger circles indicating up-regulated DEGs and smaller circles representing down-regulated DEGs. (d) Valine, leucine, and isoleucine degradation pathway in DCM hearts, expression changes of target genes and metabolites are mapped by colors: green color – statistically significant down-regulated genes, red color – statistically significant up-regulated genes, yellow color – statistically significant up-regulated metabolites, and blue color – statistically significant down-regulated metabolites.

To assess the correlation between the transcriptome and metabolome, the correlation coefficient was calculated using the Spearman method with R packages. The heat map analysis in Figure 4a shows both positive and negative correlations between various genes and metabolites, suggesting a potential influence of the transcriptome on metabolic processes.

To further investigate the relationship between DEGs and DAMs, we mapped them to the KEGG pathway database. This enabled the identification of pathways significantly enriched with both differential metabolites and DEGs. A joint KEGG enrichment analysis of the transcriptome and metabolome revealed six significant pathways (Figure 4b). Among these, the degradation of valine, leucine, and isoleucine was the most significantly dysregulated at both the gene and metabolite levels. A total of 36 DEGs and 2 DAMs were involved in this pathway’s dysregulation. Visualization of the integrated pathway-level analysis from transcriptomics and metabolomics data using Pathview showed that the expression of most genes associated with this pathway is down-regulated, including BCAT2, BCKDHA, BCKDHB, DBT, and DLD genes (Figure 4c). BCAT2 encodes branched-chain amino acid transferase, which is involved in the first reversible step of the degradation reaction, converting BCAAs to their respective branched-chain α-keto acids (BCKAs). Subsequently, BCKAs undergo irreversible oxidative decarboxylation mediated by branched-chain α-keto acid dehydrogenase (BCKDH) to produce coenzyme A (CoA) esters. BCKDH comprises four catalytic subunits: BCKDHA/E1α, BCKDHB/E1β, DBT/E2, and DLD/E3, with BCKDHA and BCKDHB encoding the key catalytic subunits of BCKDH. Our study detected an increase in NADH, indicating an inhibition of BCKD kinase activity. Along with the down-regulation of BCAA catabolic gene expression, there was a significant up-regulation of intramyocardial levels of the BCAA valine.

### 3.8 Gata3 are the key TFs that regulate valine, leucine, and isoleucine degradation

**Figure 5:**
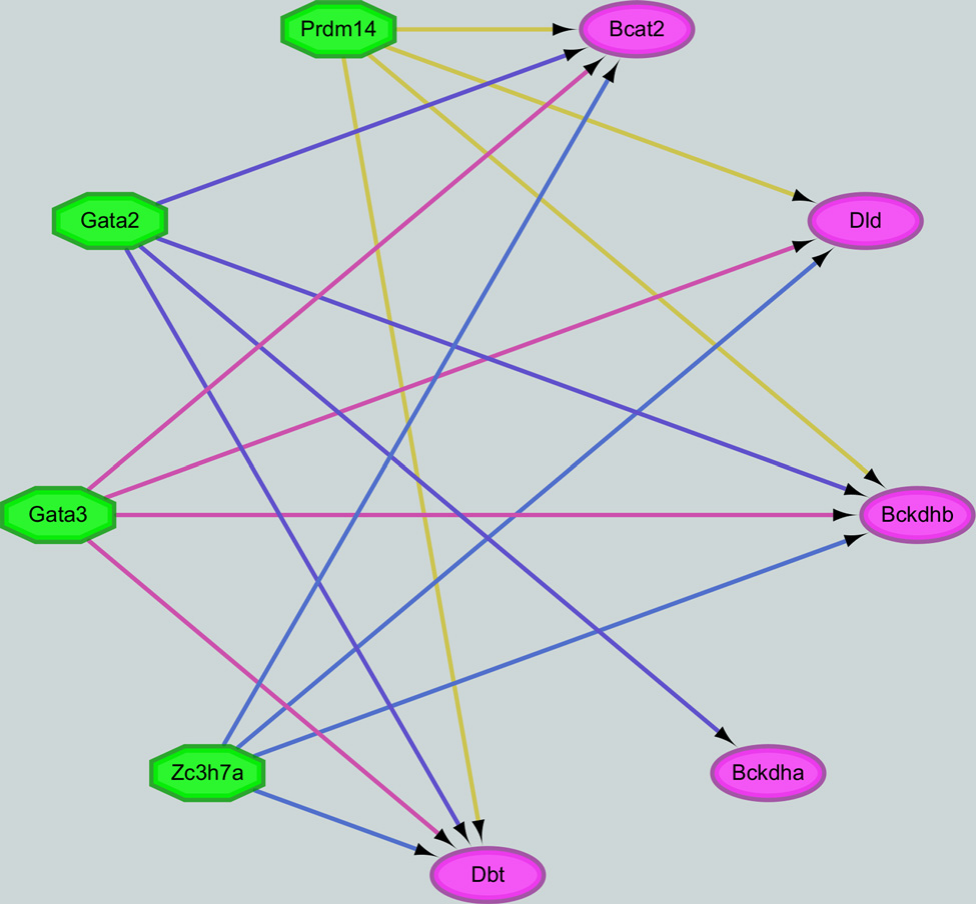
The TF-target gene regulatory network. Circles represent genes, and hexagons represent TFs.

To further understand the regulation of valine, leucine, and isoleucine degradation in DCM, key DEGs including BCAT2, BCKDHA, BCKDHB, DBT, and DLD were analyzed using the iRegulon plugin in Cytoscape software to identify TFs. A total of 40 TFs were predicted to regulate BCAT2, BCKDHA, BCKDHB, DBT, or DLD. Among these, Zc3h7a, Prdm14, Gata3, and Gata2 were predicted to regulate four out of the five key genes. Further analysis found that Gata3, detected in our RNA sequencing data, was significantly up-regulated. Consequently, Gata3 was identified as a negative regulator in the valine, leucine, and isoleucine degradation pathway, as its target genes BCAT2, BCKDHB, DBT, and DLD were down-regulated (Figure 5).

## 4 Discussion

In our study, we found that the disruption of valine, leucine, and isoleucine degradation is a critical characteristic of myocardial damage induced by diabetes, as revealed by integrated transcriptome and metabolome analysis in DCM rats. This down-regulation of BCAA degradation in diabetic hearts is likely due to the increased expression of Gata3, a TF that regulates the key enzymes involved in valine, leucine, and isoleucine metabolism, as identified using the iRegulon plugin in Cytoscape software.

BCAAs, which include leucine, isoleucine, and valine, are essential nutrients primarily obtained from the diet. They are crucial for protein synthesis and energy metabolism and serve as important nutritional signals with significant physiological and pathological effects [13]. Research in animal models and clinical studies has shown that abnormalities in BCAA catabolism are a prominent metabolic feature in cardiac diseases [13,14,15,16].

Elevated plasma BCAA levels have been observed in individuals with HF, and epidemiologically, increased circulating BCAA levels are considered biomarkers for HF and can predict adverse outcomes in these patients [17–24]. Studies in both animal models and clinical settings have also demonstrated that a deficiency in BCAA catabolism is a prominent metabolic characteristic in cardiac diseases such as compensated and decompensated HF and dilated cardiomyopathy [13,25–32].

Upon ingestion, BCAAs contribute to protein synthesis and cellular signaling. The homeostasis of BCAAs is tightly regulated, and excess BCAAs are degraded via a catabolic pathway in the mitochondrial matrix [33]. Initially, BCAAs are converted into branched alpha-ketoacids (BCKAs) by branched-chain amino acid transferases (BCATs) [34,35]. BCATs exist in two isoforms: the cytosolic form (BCATc or BCAT1) and the mitochondrial form (BCATm or BCAT2) [36]. The irreversible oxidation of BCKAs, the rate-limiting step of BCAA catabolism, is performed by the BCKA dehydrogenase (BCKDH) complex [33,37]. The BCKDH complex comprises three components: an E1 decarboxylase with α/β subunits encoded by BCKDHA and BCKDHB genes; an E2 dihydrolipoyl transacylase encoded by the DBT gene; and an E3 dihydrolipoyl dehydrogenase encoded by the DLD gene [38]. The activity of BCKDH is negatively regulated by the phosphorylation of the E1α subunit by BCKD kinase, while dephosphorylation by protein phosphatase 2Cm (PP2Cm) activates BCKDH [39]. Additionally, an increased NADH/NAD + ratio inhibits BCKDH activity [33,40]. In our study, the expression of the gene encoding BCAT2 and the subunits (Bckdha/E1α, Bckdhb/E1β, and DLD) of BCKDH was down-regulated, indicating a disruption in the transcription of key enzymes involved in BCAA degradation. Along with the down-regulation of BCAA catabolic gene expression, there was a significant accumulation of intramyocardial BCAAs, particularly valine.

The impairment in cardiac BCAA catabolism is unlikely to affect energy supply in HF *per se*, as BCAA oxidation contributes less than 5% to ATP production, even in a healthy heart [41,42]. However, animal studies have shown that disruptions in BCAA metabolism can affect cardiac function, vascular remodeling, glucose and lipid metabolism, inflammation, and tissue fibrosis [43–45].

Deficiencies in BCAA catabolism can directly impair cardiac function [29,46]. In a mouse model with inactivation of the BCKDHA gene, which encodes the E1α subunit of the BCKDH complex, a complete blockage of BCAA catabolic flux resulted in rapid and severe contractile defects in the heart [47]. Studies on myocyte-specific PP2Cm-KO mice indicate that deficient BCAA catabolism in the heart can directly impair cardiac function and accelerate pressure overload-induced cardiomyopathy [47]. Our study also observed systolic and diastolic dysfunction in DCM rats, accompanied by significant disruption in BCAA degradation, suggesting that impaired BCAA degradation may be a critical mechanism in diabetes-induced heart injury.

Inflammation is a key factor in many metabolic diseases, including diabetes, obesity, and cardiovascular diseases [48–50]. Liu et al. [51] reported that long-term high BCAA supplementation increased BCKA levels, inflammation, and tissue fibrosis (liver and kidney) in rodents. Additionally, a significant relationship was found between isoleucine and IL-6 in patients with metabolic syndrome, suggesting that isoleucine may be a potential predictive biomarker for the pro-inflammatory state of metabolic syndrome [52]. In our study, we observed a mild increase in inflammatory cells in the myocardial interstitium, especially within the capillaries, compared to the control group.

The disruption of valine, leucine, and isoleucine degradation has been reported to affect glucose and lipid metabolism as well as mitochondrial function and structure. Several animal studies have shown that the accumulation of BCAAs and their metabolites can directly inhibit pyruvate dehydrogenase (PDH) activity, leading to a significant reduction in glucose uptake and oxidation [53–55]. Specifically, high BCAA levels disrupt mitochondrial pyruvate (the end product of glucose oxidation) utilization by inhibiting PDH activity [54]. Regarding lipid metabolism, the metabolism of BCAAs and fatty acids appears to be functionally related, as previous studies have shown that KLF15 regulates both the BCAA catabolic pathway and PPARα activity, which are involved in fatty acid metabolism in cardiomyocytes [13,56–59]. Additionally, studies in animal models have indicated that E1α knockdown, which suppresses fatty acid oxidation, is associated with changes in the expression of molecules that regulate fatty acid metabolism [60]. Research on non-alcoholic fatty liver disease (NAFLD) and breast cancer has also shown that the loss of BCAA catabolism significantly contributes to mitochondrial dysfunction [61–64]. In our study, alongside the significant disruption of BCAA catabolism, GO pathway analysis revealed a significant enrichment of DEGs in terms related to mitochondrial structure or biological processes. These included the mitochondrial inner membrane, mitochondrial matrix, mitochondrial large ribosomal subunit, mitochondrial respiratory chain complex I, assembly of mitochondrial respiratory chain complex I, tricarboxylic acid cycle, ATP metabolic process, and mitochondrial electron transport. Most of the genes in these categories were down-regulated, indicating impaired mitochondrial function and structure. KEGG pathway analysis of DEGs revealed significant enrichment in pathways related to carbon metabolism, the citrate cycle (TCA cycle), pyruvate metabolism, glyoxylate and dicarboxylate metabolism, propanoate metabolism, oxidative phosphorylation, and fatty acid metabolism. Furthermore, the metabonomic analysis indicated that the DAMs were predominantly enriched in sets of metabolites such as peptides and analogs, fatty acids and conjugates, carbohydrates and carbohydrate conjugates, and glycerophosphocholines. These findings suggest that the disruption of branched-chain amino acid metabolism occurs concurrently with abnormalities in glucose and lipid metabolism and mitochondrial dysfunction. However, the causal relationship among these factors requires further investigation.

GATA-3, part of the GATA family of TFs, plays a crucial role in regulating hematopoietic stem cells and their derivatives [65,66]. Studies in mGATA3KO mice have shown that the deficiency of GATA3-positive macrophages can improve cardiac function following myocardial infarction or pressure overload hypertrophy [67–69]. In our study, GATA3 was significantly up-regulated and identified as a TF regulating the key enzymes involved in the degradation of valine, leucine, and isoleucine. GATA3’s involvement in amino acid metabolism regulation is supported by studies in breast cancer, where Glutaminase 2 (GLS2), transcriptionally regulated by the estrogen receptor co-factor GATA3, is more common in hormone receptor-positive tumors [70,71]. Furthermore, research on clear cell renal cell carcinoma has demonstrated the tumor growth-inducing functions of the AMPK–GATA3–ECHS1 pathway. In this pathway, BCAA accumulation, increased by ECHS1 down-regulation, acts as a promoter of mTORC1 and *de novo* fatty acid synthesis, thereby enhancing cell proliferation [72,73]. Our study predicted GATA3 as a negative regulator of the genes encoding key enzymes in the degradation of valine, leucine, and isoleucine.

In summary, our results indicate that the disruption of BCAA degradation is a critical characteristic of myocardial damage induced by diabetes. This disruption occurs concurrently with abnormalities in glucose and lipid metabolism, damage to mitochondrial structure and function, significant cardiac dysfunction, cardiac remodeling, and a mild increase in inflammatory cells in the myocardial interstitium, particularly within the capillaries. GATA3 was identified as a TF regulating key catalytic enzymes involved in branched-chain amino acid metabolism. While the causal relationship between the disruption of BCAA degradation and other factors, such as cardiac dysfunction, abnormalities in glucose and lipid metabolism, and increased inflammatory cell infiltration in DCM, requires further investigation, our findings provide valuable insights for future studies on DCM.

## Supplementary Material

Supplementary material

Supplementary Table 3

Supplementary Table 4

Supplementary Table 5

Supplementary Table 6

Supplementary Table 7
