# Supplementary material for: Disruption of BCAA degradation is a critical characteristic of diabetic cardiomyopathy revealed by integrated transcriptome and metabolome analysis
Source: Open Life Sci. 2024 Dec 31;19(1):20220974. doi: 10.1515/biol-2022-0974 (PMC11736389; doi:10.1515/biol-2022-0974)
Supplement: Supplementary material [file biol-2022-0974-sm.pdf]

Supplementary material

Table S1: Echocardiographic analysis of DCM rats

Parameters	Control Group	Model Group	*P*-Value
Ivsd(mm)	1.46 ± 0.04	1.41 ± 0.21	0.6444
LVIDd(mm)	7.97 ± 0.8	8.02 ± 0.66	0.8829
LVPWd(mm)	1.48 ± 0.17	1.20 ± 0.12	0.0046
IVSs(mm)	2.10 ± 0.34	1.88 ± 0.41	0.3123
LVIDs(mm)	4.66 ± 0.48	5.04 ± 0.53	0.1971
LVPWs(mm)	2.40 ± 0.21	1.95 ± 0.24	0.0034
HR (bpm)	408.83 ± 30.38	279.5 ± 17.05	<0.0001
LVEDV(mL)	1.13 ± 0.31	1.15 ± 0.26	0.8957
LVESV(mL)	0.26 ± 0.08	0.32 ± 0.09	0.1974
SV (mL)	0.87 ± 0.24	0.83 ± 0.19	0.7172
EF (%)	77.55 ± 2.38	72.74 ± 3.54	0.0143
FS (%)	41.45 ± 2.28	37.34 ± 2.82	00129
MV E/A Ratio	1.20 ± 0.13	1.52 ± 0.25	0.0160
MV Vel E(cm/s)	86.22 ± 11.24	73.51 ± 9.45	0.0402
MV Vel A(cm/s)	72.22 ± 11.96	49.48 ± 9.86	0.0021
MV Dec Time(ms)	28.67 ± 3.27	38.25 ± 4.62	0.0010
IVRT(ms)	15.33 ± 2.34	26.75 ± 3.77	<0.0001
MV e'(cm/s)	5.25 ± 1.24	3.38 ± 0.73	0.0040
E/e'	16.83 ± 2.76	22.55 ± 5.43	0.0369

Table S2: The class of the differentially altered metabolites

Class	Numbers of DAM	Percentage (%)
Amino acids, peptides, and analogues	21	18.92
Fatty acids and conjugates	17	15.32
Carbohydrates and carbohydrate conjugates	6	5.41
Glycerophosphocholines	5	4.50
Bile acids, alcohols and derivatives	3	2.70
Fatty acid esters	2	1.80
Glycerophosphoglycerols	2	1.80
Glycerophosphoserines	2	1.80
Hydroxyindoles	2	1.80
Benzoic acids and derivatives	2	1.80
Others	49	44.14

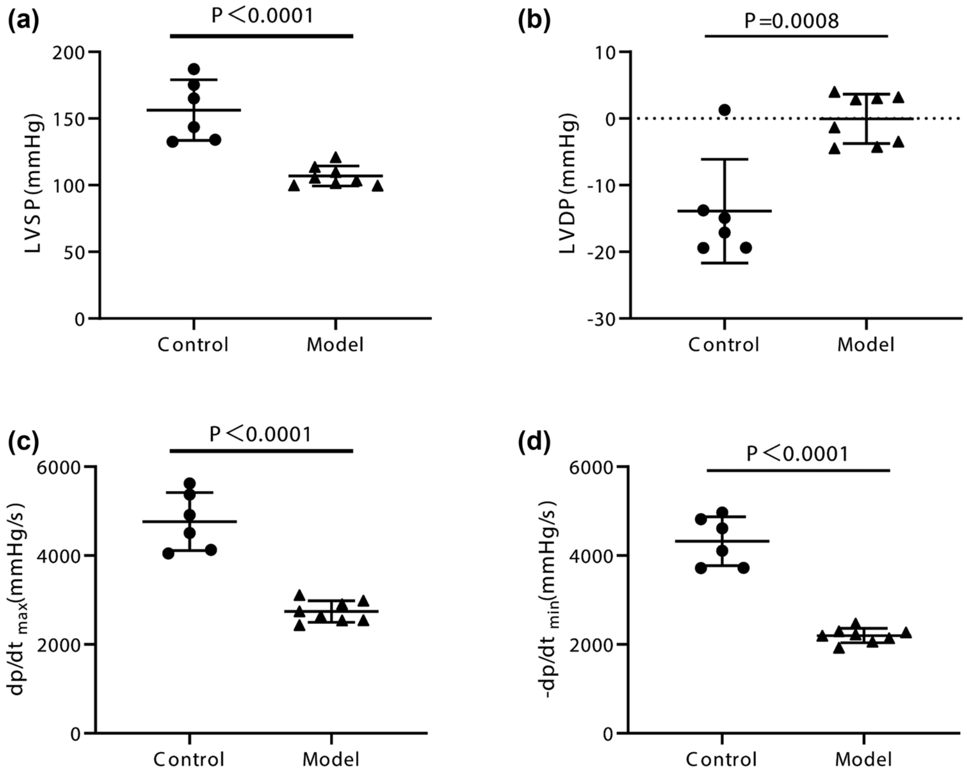

Figure S1: DCM has a negative impact on both diastolic and systolic function, leading to impaired cardiac performance by hemodynamics analysis, (a–d), Measurement of left ventricular systolic pressure (LVSP), left ventricular diastolic pressure (LVDP), maximum rate of pressure rise during systole (+dp/dt_{max}) and maximum rate of pressure decline during diastole (−dp/dt_{min}).

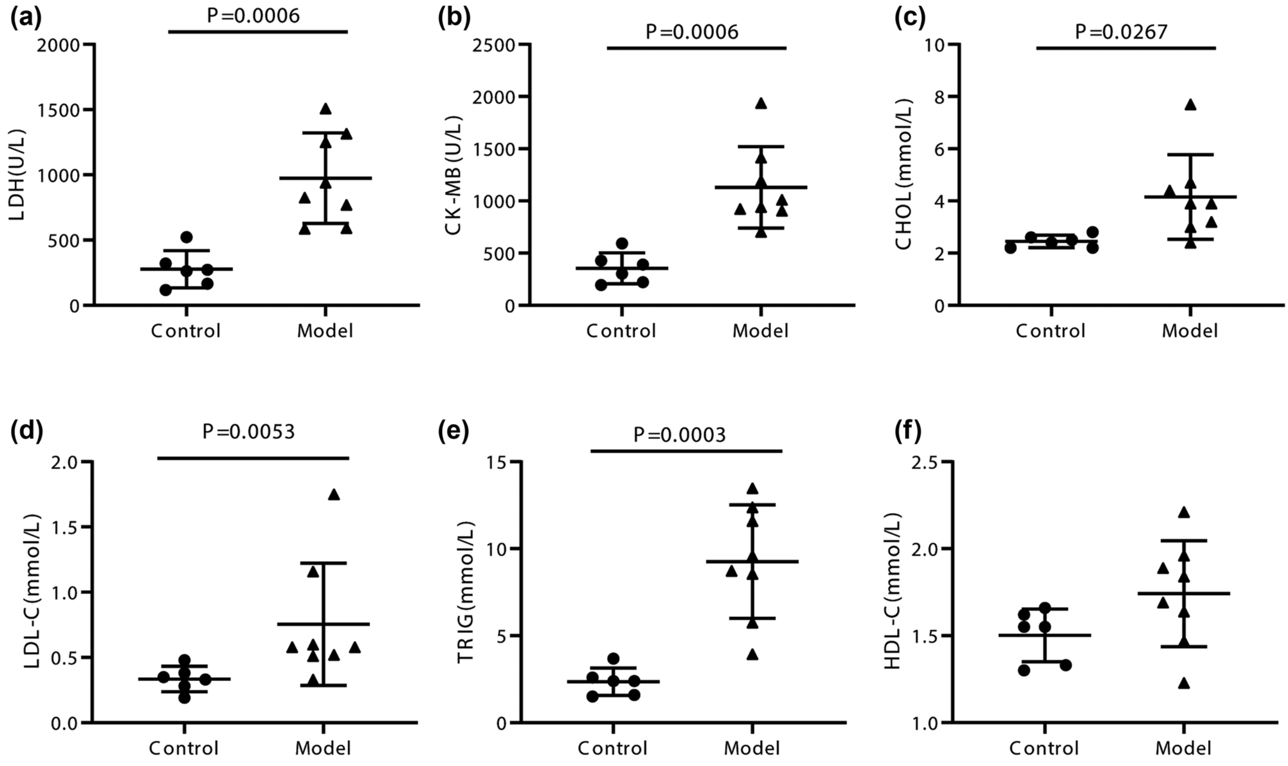

Figure S2: Biochemical parameters and lipid profile in serum, (a and b), serum cardiac injury markers LDH (a) and CK-MB (b), (c–f), the results of serum lipid levels.

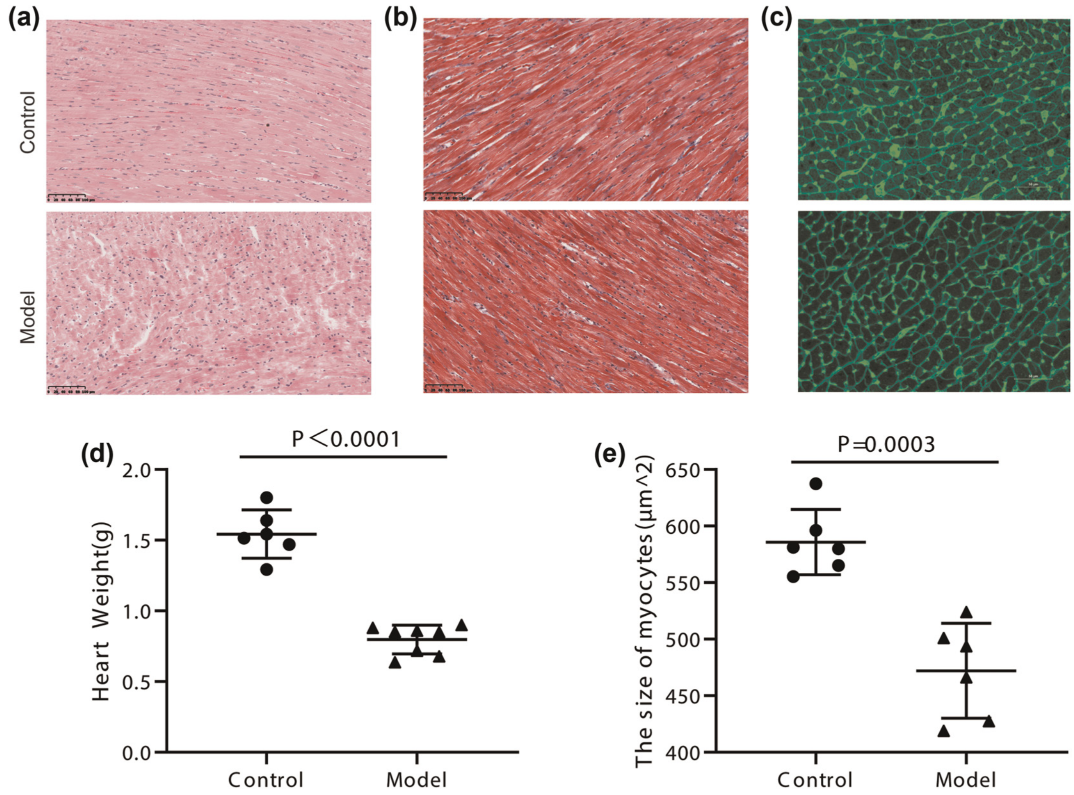

Figure S3: Diabetes change the morphology of myocytes. (a), Representative images of HE staining (200×), (b), Representative images of Masson staining (200×), (c), Representative images of WGA staining (400×), (d) the heart weight, (e), the calculated myocyte cross-sectional area.

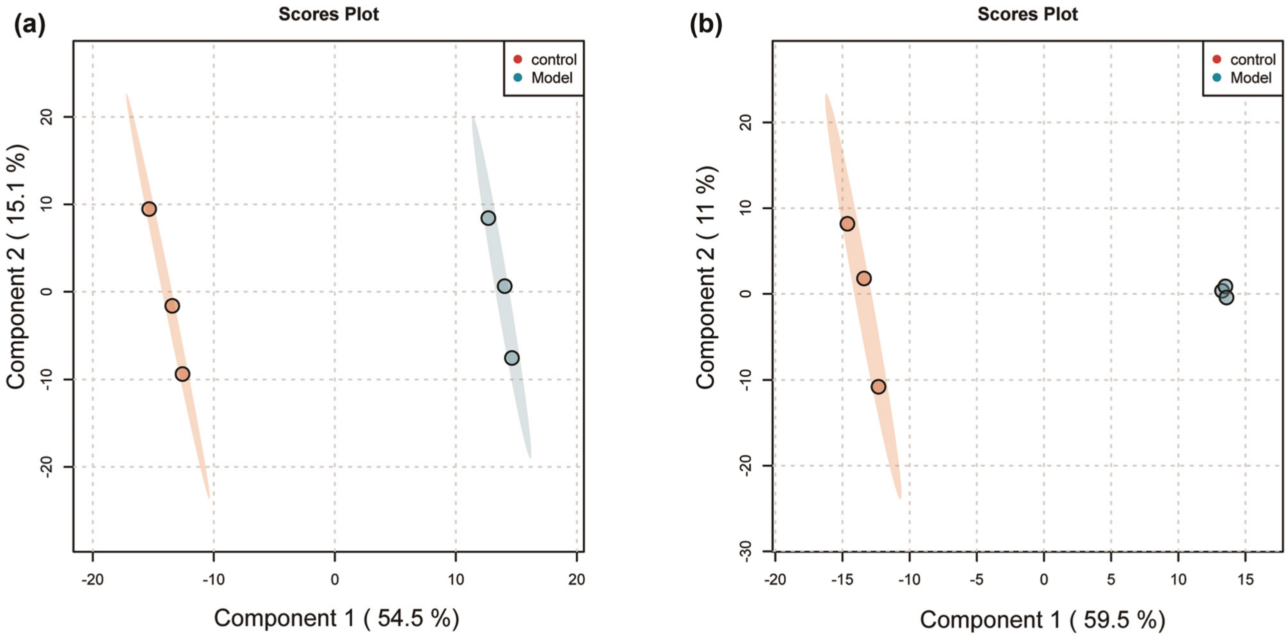

Figure S4: Partial least squares discriminant analysis (PLS-DA) score plots. (a) Positive mode, (b) negative mode.
